# Where are the students? A close reading of priorities and silences in scholarly and public debates on VCE English (1990–2021)

**DOI:** 10.1007/s13384-022-00543-2

**Published:** 2022-08-05

**Authors:** Allayne Horton, Larissa McLean Davies

**Affiliations:** Melbourne Graduate School of Education, University of Melbourne, Melbourne, Australia

**Keywords:** Senior English, VCE (Victorian Certificate of Education), Subject English, Narrative scoping review

## Abstract

Debates about subject English in Australia are often conducted through the senior years curriculum. In light of the anticipated interest in the new Victorian Certificate of Education (VCE) English study design released in 2022 to be implemented in 2023, this paper outlines the current state of research on the VCE English subject by mapping areas of interest, types of evidence and gaps in research. The authors utilise a hybrid approach of narrative scoping review to identify methodological and thematic trends in the scholarly literature, and intersecting professional and media discourse on VCE English from 1990 to 2021. Finding that the student experience and the enacted curriculum have been largely elided, the paper identifies fresh lines of inquiry into VCE English and advocates for new discussions around scholarly interest and approaches to senior secondary English in Australia.

## Background

The Victorian Certificate of Education (VCE) English subject is a key tenet of Victorian students’ senior secondary education. In order to receive their VCE, students must complete at least three units from the English subjects; the most generalist and populist of these offerings is the VCE English course, with over 41,000 students sitting the exam in recent years (VCAA, 2021a). The Victorian Curriculum and Assessment Authority (2014) (VCAA) recently undertook a review of the VCE English curriculum (see VCAA, 2014)  including “the rationale for and focus of the study”, organisation of “knowledge and skills” and assessment components (VCAA, 2021b), resulting in a new Study Design released publicly in March, 2022 (see VCAA, 2022). Through various iterations since its inception in 1990,^1^ VCE English has been a continuous magnet for scholarly, professional and media debate. Thus, it is unsurprising that comments on teacher agency as well as the knowledge and skills contained in the 2023 Study Design have already begun to emerge (Carey, 2019).

The topic of VCE English offers an enlightening microcosm for curriculum development and reception in Australia. In short, the 1980s marked the beginning of Victoria’s consolidation towards one matriculating pathway for senior secondary students. In 1982, the newly elected Labor government, emboldened by a reformist agenda and later the Blackburn Report (1985), which recommended a broadening of the curriculum in line with increasing student participation in Years 11 and 12, took action to reshape post-compulsory education in Victoria (Howells, 2003/2019). These sweeping changes became the VCE: the standardised certification for all government and non-government schools, colleges and Technical and Further Education (TAFE) institutions. Under the Victorian Curriculum and Assessment Board (VCAB), VCE English was one of three core subjects to be implemented in 1990 alongside maths and Australian Studies, replacing the multiple and divergent Englishes taught across the state (Gill, 1994). Through various iterations in the following three decades, VCE English has maintained its status as a central feature of the VCE qualification as well as an area of keen public and scholarly interest.

This paper utilises elements of scoping and narrative review to lay the foundations for fresh and targeted research on VCE English by opening new conversations about preoccupations and underexplored areas. More broadly, this research contributes to investigations into the ways that senior subjects, in high-stakes contexts, shape and define disciplinary fields. The review responds to the following framing questions:

(i) What methodological trends are dominant in the scholarly work on VCE English?
(ii) What thematic trends are dominant in scholarly, media and professional publications on VCE English?
(iii) What underexplored methodologies and focal areas require attention to understand the discipline in our contemporary moment and what it may become?

After mapping trends and highlighting silences, the paper concludes with a meditation on why the scholarship on VCE English has ossified in the manner identified and calls for action on pertinent gaps in knowledge regarding the enacted curriculum and student experience.

## Methodology

Before detailing the methodology and methods employed, it is important to foreground our own situatedness in the histories and present enactments of VCE English. Author One teaches and researches VCE English, her emergent scholarly work pays particular attention to theories of affect and the classroom interface. Author Two is a teacher educator who has written extensively on subject English in Australia, with a particular focus on the teaching of literature and the development of teacher knowledge. It is from this positionality that we approach VCE English as a space characterised by our lived experiences of dynamic classroom moments, and as a scholarly curiosity that speaks to broader historical and ideological concerns.

To map preoccupations and silences in scholarly and public debates on VCE English, we synthesised elements of narrative and scoping review (Ladha et al., 2019; Pham et al., 2019). Although taking a hybrid approach, the paper can be characterised as predominantly narrative in nature, as it was determined that this narrative description or commentary would be the most appropriate way to frame the findings to foreground rationales for future research (Ferrari, 2015).

The selection criteria and organisation of the literature were informed by a scoping review approach. This approach was selected as scoping reviews allow researchers to map the concepts or characteristics of broader fields of inquiry (Munn et al., 2018) and identify “key concepts, types of evidence and gaps in research” (Horsley, 2019, p. 55). Others have similarly highlighted the important “reconnaissance” work scoping reviews perform (Peters et al., 2015, p. 141), particularly their potential to enrich more fundamental understandings of a field by “report[ing] on the types of evidence that address and inform practice” (Peters et al., 2015, p. 142). This is important for research on VCE English, which will soon receive renewed scholarly interest on the latest iteration of the curriculum published in 2022, but has yet to be the subject of systematic literature review. It should be acknowledged, however that this approach is necessarily narrow: it excludes much of the grey literature (e.g. blog posts, teaching resources) or scholarly work that is not explicitly focussed on the VCE English space.

We began the review by searching for peer-reviewed publications in the ‘A + Education’ and ‘Education Database (ProQuest)’ databases with the following key terms: ‘[VCE OR Victorian Certificate of Education] AND [English]’, limiting to Australian work between 1990 and 2021. These searches yielded 134 and 67 results respectively. Duplicates were removed and the remaining results were screened and selected for inclusion based on a close or sustained focus on VCE English, as opposed to a fleeting mention or exploration of alternative foci such as other senior secondary subjects or VCE English Language or Literature. From this screening process, 17 scholarly publications were selected for inclusion. Additional texts on VCE English referenced across these publications by key authors in the field were also included, bringing the total number of scholarly publications included in the paper to 23. Additional searches were then undertaken with the same key terms and constraints in the aforementioned databases as well as the ‘Australia and New Zealand Newsstream’ database. Media articles or comments, and papers from the Victorian Association of English Teachers (VATE)’s professional journal Idiom were then selected for inclusion based on intersections with identified thematic trends. As the review was interested in the Victorian senior English context, it was determined that a focus on the professional journal of Victorian English teachers, Idiom*,* was an apt way of sourcing veritable and representative voices or areas of interest of relevant teachers. 18 papers from Idiom and 12 articles or comments from media outlets were included in the narrative synthesis.

We utilised a close reading method in tandem with a complementary ‘reading for affect’ (Berg et al., 2019) to identify methodological and thematic trends. Close reading is a cornerstone of literary studies and is defined as “a technically informed, fine-grained analysis” (Smith, 2016, p. 59) that pays attention to recurrences of key words and concerns to identify trends (for example, see McLean Davies & Sawyer, 2018). As suggested by Berg et al. (2019), this method may be fruitfully coupled with a reading which employs concepts of affect and emotion “as sensitizing concepts in the analysis and interpretation of language and discourse” (p. 58). We took this up adjunctively by identifying emotional vocabulary and authorial tones.

Given the considerable overlap between methodologies and thematic trends (i.e. teacher interviews are most often focussed on pedagogy or teachers’ perspectives), the paper has been organised around methodological and thematic attachments. These focus attention on the often limiting and repetitive nature of methodological and thematic fixtures, urging scholars to challenge the ideological or perceived practical limitations of research in VCE English.

## Discussion

The following methodological and thematic pairings were found to be dominant in the scholarship on VCE English: curriculum histories concerned with external interference, text list analysis and ideological concerns, critical readings of examination documents for authenticity and equity, and interviews with and about teachers. Reviewing key areas where trends in research mirror concerns in the media and Idiom*,* we gesture towards what continues to be overlooked and how these limitations might be overcome with future research.

### Curriculum histories concerned with external interference

This section of the review presents an overview of scholarly and public concerns with and about curriculum change and how the notion of external interference has framed VCE English curriculum matters.

Scholars have repeatedly returned to the story of the original VCE English curriculum in the late 1980s to gesture towards lost potential for a socially democratic and authentic version of senior English. The original course is characterised in these histories as having a “democratic impulse” (McLean Davies et al., 2017a, p. 149), a construction and application of English for authentic purposes, and as being a more equitable, “doable, purposeful, [and] relevant” course for senior English students (Hayes, 1997, p. 83). The optimism aflame in the moment of its inception, of what a progressive model of VCE English might be for students and teachers, reemerges as nostalgia or melancholy in these accounts. What also emerges in these accounts is the perceived agency of political forces to constrain the lofty goals of the course (Doecke & Hayes, 2000; Gill, 1994; Huggard, 1997). Given that the VCE course emerged in the late 1980s from the imaginary of a newly elected Labor government emboldened by a reformist agenda to reshape post-compulsory education in Victoria (Howells, 2003/2019), it is perhaps unsurprising that conservative commentators took issue with the curriculum.^2^ Conservative commentaries about the “very unsatisfying and unsatisfactory” nature of the original Study Design (Bantick, 1992, p. 11) are likewise mirrored by media headlines of this period curated by Gill (1994) such as “VCE and the red peril” (p. 101) and “Curriculum change and the barbarians at the gates” (p. 104). These contemporary accounts and curriculum histories repeatedly gesture towards the power of politically conservative voices in the media and in universities (Teese, 2000) to constrain the egalitarian potential of the VCE English course (Gill, 1993, 1994).

Furthermore, accounts of the origins and implementation of the original VCE English course in scholarly and Idiom publications evidence a sense of disappointment around the loss of teacher agency in curriculum design. As the convenor of the English Field of Study Committee responsible for developing the original curriculum, Howells’ reflective accounts (2002,2003/2019) point to a sentiment echoed in other reviews of the development of VCE English (Doecke, 2019; Hayes, 1997, 2015, 2018, 2020; McLean Davies et al., 2017a, 2017b) that this period exemplifies a lost gold standard of teacher partnership in curriculum development; it “shows a group of teachers in action developing a design and implementing it. It shows what teachers can do'' (Howells, 2003/2019, p. 68). Likewise, Hayes (2015) fondly recalls the “grand idealism” (p. 1) of the original VCE English curriculum as the pinnacle of teacher inclusion and professionalism in curriculum development. These authors lament that now “[t]eachers have become ‘functionaries’ rather than professionals, they deliver a curriculum that they do not ‘own’” (Howells, 2003/2019, p. 69). This idea of a regression in teacher partnership is a key focus of Idiom’s (2019) issue, ‘Conversations about the original VCE English Study Design’. Precipitated by VATE’s commissioned study on the initial curriculum (Hayes, 2015), this issue illustrates a clear coalescence between the voices of scholars and educators, reinforcing key tropes of socially democratic objectives constrained by political forces (Doecke, 2019) and lost teacher participation in curriculum development (Hayes, 2019; Howells, 2003/2019; McRae & Maher, 2019; Osmotherly et al., 2019; Reid & Doecke, 2019). More recently, in what might be seen as progress in teacher partnership in curriculum development, the VCAA’s Study Review Panel for the latest iteration of the Study Design included representatives from all school sectors, and the VCAA invited feedback on the draft curriculum from educators in 2021 via an opt-in Consultation Register on their webpage (VCAA, 2020). The extent to which teachers’ views and feedback were utilised, however, remains unknown.

The continued preoccupation with the original VCE English Study Design points towards scholarly and professional uncertainties with consultation processes in contemporary curriculum development and concerns over the influence of neoliberalist imperatives impacting on subject English, reducing the scope of texts considered legitimate for study and legitimate readings of these texts (Yandell et al., 2020) as well as constraining teacher identity and pedagogical agency (McKnight, 2016). It is against this backdrop that new scholarly considerations of VCE English are emerging that reconsider curriculum through new frames of reference, such as the cultivation of morality (Zhou, 2020), or the legitimisation of certain forms of knowledge and the authentic possibilities of ‘youth literacies’ (Bacalja, 2021). As scholars and teachers, therefore, look towards the implementation of the latest VCE English Study Design, these historical accounts and emerging curriculum-focussed work beg the questions: what does successful curriculum change in VCE English look like? How successful can curriculum change be when external forces appear more agentive than internal stakeholders? The answers to these questions remain pertinent and opaque. Generative future research might engage with the complex dynamics and influences of competing ideologies and actors in contemporary VCE English curriculum design, including the influence of teachers’ contributions in consultation processes; the pressures of increasingly neoliberal educational imperatives and privileging of the science, technology, engineering and maths disciplines; as well as the agency of post-traumatic (or post-pandemic) political climates.

### Text list analysis and ideological concerns

As they have occupied a central position in the VCE English classroom, so too have set texts received much scholarly and public attention. This section begins with a brief overview of the practical requirements of text selection in VCE English as background, then tracks interest in the ‘right’ texts for VCE English through ideological concerns over nationality, modality, representation and community sensibilities in scholarship, Idiom and the media.

The VCAA regulates which texts are studied in VCE English through the annual Text List proposed by the Text Advisory Panel. Previous study designs have required schools to select four texts from this list for implementation with Units 3 and 4 of the VCE English Study Design, with at least one Australian text and no more than one multimodal text (VCAA, 2018). The 2023 Study Design dictates that five texts be selected from this list, maintaining only one multimodal text may be selected, but adding an additional layer of specificity with regards to the Australian text, that it be “by an Aboriginal and Torres Strait Islander or other Australian author or creator that directly explores Australian knowledge, experience and voices” (VCAA, 2022, p. 21). Furthermore, the introduction of ‘mentor texts’ as a core feature of the 2023 Study Design (VCAA, 2022) may signal a change in text selection practices and the utilisation of texts in classrooms. There is a strong interplay between the construction of this text list and public debates on texts in VCE English, as explored below.

Scholarship on texts in VCE English has tended to focus on critical readings of policy documents about texts and text lists, extrapolating upon the implications for practice through quantifiable patterns. This is informed by the understanding that texts serve as proxies for literary knowledge in subject English (Yates et al., 2019), thus, ideological debates about texts in VCE English speak to broader concerns about the nature of the subject. The imperative to cultivate a national literary identity through Australian literature (Doecke et al., 2011; McLean Davies et al., 2013, 2017b, 2021a) is one key ideological concern played out in studies of VCE English texts. This is exemplified by Patterson’s (2012) review of Victorian Curriculum and Assessment Authority (VCAA) examination data from 2001 to 2010, which revealed that few students wrote on Australian texts in their VCE English exam. To what extent this is indicative of a broader resistance to Australian literature as a continued manifestation of ‘cultural cringe’ (McLean Davies et al., 2021b), or points towards other matters of the high-pressure senior secondary context, or situated text selection or pedagogical priorities, is largely unknown. Also exploring a quantifiable aspect of texts in VCE English through VCAA examination data, Weaven and Clark (2009) gesture towards concerns around equity and modality when identifying a correlation between higher scores and students writing on poetry, although remain primarily focussed on the inherent features of poetic texts rather than how these might be activated by students and teachers.

Further critical readings of VCAA documents undertaken by Bacalja and Bliss have taken up cognate concerns with disrupting the centrality of the English canon to embrace more diverse modalities and narratives in VCE English. Drawing upon data from VCE English text lists between 2010 and 2019, Bacalja and Bliss’s Report on Trends in Senior English Text-Lists (2018), and associated scholarly outputs (2019a, 2019b, 2020), highlight a continued prioritisation of British texts, and marginalisation of Indigenous authors, narratives about non-heteronormative figures and of multimodal or pop-culture texts. Although this critical analysis of the texts and narratives dominating the VCE English text list in the past decade highlights important cultural and ideological shifts in the discipline, allowing for cases to be made for a more diverse and inclusive text list, it is only part of the picture. As Bacalja and Bliss acknowledge, “[s]elected texts do not constitute the curriculum in and of themselves—they must be negotiated between teacher and students, and in that negotiation the views and values of individuals impact upon the way the texts are received” (2018, p. 6). This is what is sorely missing from the scholarship on texts in VCE English: how and why are students and teachers in various classroom contexts selecting and taking up different texts—how are matters of modality and representation negotiated? Sustained ethnographic or in situ research is needed to gain a greater understanding of how texts are selected and mediated in praxis, how they interact with students, teachers, curriculums, classroom spaces and other situated actors. Author Two’s current project with the Stella Prize, in terms of observing how representationally diverse Australian texts are taken up by teachers and students in the classroom, is beginning to inform this gap in the literature.

Concerns with modality, diversity and engagement in VCE English have also played out in politicised media discourse. Where the subject has sought to embrace more multimodal or popular text types, this has been met with suspicion and anger in conservative media circles, demonstrating the enduring belief that the English canon ought to be central to subject English (Donnelly, 2007, 2018). The swift rescindment of Briggs’ graphic novel *When the Wind Blows* on the 1992 text list in response to public furore is one key example of this (McLean Davies et al., 2017a, 2017b). Likewise, the ‘English Lite’ controversy following the draft 2006 Study Design, which proposed a reduction in the number of compulsory novels, saw the reemergence of a familiar trope of conservative outrage at the “ludicrous” (“English Lite a tragedy for students”, 2005), fundamental and pernicious simplification of VCE English (“VCE English ‘dumbed down’”, 2005).

More recently in the media concerns over texts in VCE English have emerged alongside the ‘trigger warning’ debate. Bringing community sensibility to the fore and prompting the VCAA to release new text selection policy documents detailing the need for texts to “meet community standards and expectations” (VCAA, 2018), students were quoted as feeling “traumatised” by the “constant depressing messages” in their set texts (Cook, 2017a, 2017b). Although some emotional dimensions of personal writing in the VCE English classroom have been considered in past research (Etheredge, 2003) and emerging scholarly work on texts which represent emotionally or experientially ‘difficult’ knowledge in the VCE English classroom is beginning to probe the area of student and teacher emotion with provocative texts (McLean Davies & Buzacott, 2021), scholars would be well served to attend more explicitly to the agency of community sensibilities as they interplay with difficult knowledge and students’ affective responses to texts in the VCE English classroom. The affective and personal dimension of engaging with VCE English texts, particularly from the students’ perspective, remains a complex and under-researched area.^3^ Author One, in her current research investigating the affective dimensions of literary texts in situ with teachers and students in VCE English classrooms, is seeking to inform this gap in knowledge, made even more relevant by the reintroduction of a ‘personal’ approach to reading and writing foregrounded in Unit 1 of the 2023 Study Design (VCAA, 2022).

### Critical readings of examination documents for authenticity and equity

Given the inextricable linkages between the VCE and students’ final educational achievement scores, assessment has and will continue to be a key trigger for debate on VCE English. Concerns with inauthenticity and inequity, in particular, repeatedly converge around the assessment schema for VCE English, as this section of the review will argue.

Scholarly and professional curriculum histories often mourn the loss of the assessment schema developed to complement the original curriculum (Doecke, 2018; Howells, 2003/2019; McLean Davies et al., 2017a, 2017b). The modified assessment shroud, which resulted from Brown and Ball’s (1992, cited in Timmins, 2002) report on the Common Assessment Tasks and aforementioned political forces, was seen by many involved in the original curriculum design as creating a mangled “Frankenstein’s monster” (Hayes, 1997, p. 68) of a course. Focal points in historical accounts of the shift in balance from internal to external assessments have highlighted excessive student and teacher workloads and challenges authenticating student work identified by the ‘VCE Review’ (State of Victoria, 1997; Timmins, 2002). Indeed, this concern with authenticating work and creating authentic learning tasks, as well as reinforcing inequities in the educational system continue to be key concerns surrounding assessment in VCE English (Gill, 2002; Hayes, 1997; Macdonald, 1995; Radford, 2015).

For many in the media, VCE English *is* the end of year examination. This is evidenced by familiar headline tropes preceding the exam each year: “Steps to doing well on the VCE English Exam” (Heffernan, 2019) and proceeding the exam: “No droning on as VCE exams kick off with ‘no surprises’ English test” (Carey & Robinson, 2020), “Jitters eased as VCE students sail through ‘very fair’ English exam” (Carey & Heffernan, 2021). Most often, in keeping with entrenched patterns, responses in the media have been derisive, characterising the exam as absurd: “Giant watermelon in VCE English exam confuses and amuses students” (Cook & Jacks, 2016), “Students analyse giant pile of rubbish on VCE English exam” (Cook & Cowie, 2017); overtly progressive: “VCE English exam triggers Greens party propaganda concerns” (Cook, 2017a, 2017b); or inequitable: “VCE English exam catered to the experiences of affluent city students” (Anonymous, 2018). Past studies have identified media motifs in constructions of VCE English (Gill, 1994), but no contemporary work of this nature, nor focus on framings of the final examination have been undertaken. Given the consistent attention the media gives to the VCE English exam, a more comprehensive and contemporary analysis of headline trends would complement histories of political influence in VCE English (for example, Doecke & Hayes, 2000).

In scholarship, similar fixations with the end of year examination have precipitated work focussed on the intended curriculum as read through examination papers and policy documents. Parallel to the work on texts in VCE English, this research constructs a view of the subject almost exclusively on documentary analysis, reinforcing the public conception that VCE English is not 2 years of teaching and learning, rather, it is a three hour exam. Patterson’s (2008) comparative analysis of senior English examination papers across Australia points towards the importance of examiner’s reports in shaping what happens in VCE English classrooms, but ultimately concludes that a documentary approach is fundamentally limited: “it would take sustained research with cohorts of teachers in classrooms to understand this issue” (p. 321). Likewise, opining on the “optimal English examination” (2010, p. 65), McCurry’s comparative readings of examination papers in VCE English and other senior secondary English contexts in Australia reveal considerable diversities in the language and content of the examinations, but this documentary analysis is not able to offer insights into how these diversities create different paradigms of senior English in praxis, or affect students and teachers differently. Later in Idiom*,* with cursory attention to how this might change the Study Design and be implemented in classrooms, McCurry again urged that future English exams be less predictable, more authentic and more impromptu (2012). Revisiting VCE English and other Australian senior secondary examination papers and reports as data sources, Anson (2017) posited significant correlations between cultural capital and success in subject English. Again, like so much of the research on VCE English, this work elides classroom praxis by making—albeit highly informed and generative—assumptions about how students are positioned as students of VCE English by examination papers alone. One wonders how a more authentic and equitable examination/assessment overlay for VCE English can be realised when the student experience of the examination or the ways in which the examination shapes teaching and learning in situ have yet to be studied. Given that one key change signposted in the 2023 revised curriculum is a “framework of ideas” area of study where students will be encouraged to write in diverse genres and forms (VCAA, 2021a, 2021b, 2022), new concerns with equity and authenticity in assessment and the end of year examination will come to the fore and require attention that moves beyond documentary analysis.

### Interviews with and about teachers

Although one can easily find a plethora of teachers’ voices in Idiom*,* in the scholarly literature VCE English teachers’ voices are far more difficult to locate due to the sheer lack of qualitative and in situ research undertaken in this context. This section reviews qualitative work on VCE English encapsulated by teacher interviews and pedagogical concerns.

Jordan’s (2008) study of digital tools in the VCE English classroom is presently one of only a handful of peer-reviewed, published studies to engage in situ qualitative methods in this space. Partnering with a single VCE English teacher for interviews and limited observations, Jordan explored the utility of a discussion forum for a novel study in Units 1 and 2 English. The study found that asynchronicity afforded new opportunities for teaching and learning; however, its focus was teacher satisfaction, rather than how the technology could be utilised in critical or creative ways, and students were only spectral presences. Thus, a pertinent gap in knowledge herein remains: how do digital tools and programmes mediate or interplay with VCE English texts and curriculum? How might programmes and technologies shape learning interactions, engagements and understandings? As Wynne presciently notes in an Idiom paper exploring literature and online learning, “delivering curriculum to a student in the comfort of their own home [is a] dystopian vision [that] is worth an article of its own” (2000, p. 39). Given recent experiences with COVID-19, which have forced students and teachers into new and enduring paradigms of technology-mediated learning, these questions about digital technologies in VCE English are even more pertinent.

Other qualitative studies have sought to engage with VCE English teachers’ perspectives on texts, pedagogies and professional identities. Weaven and Clark’s (2013) consideration of the role and value of poetry in VCE English found that teachers’ anxieties around exam performance and personal unfamiliarity with poetry shaped their text selection decisions. Revisiting the same interview data, Weaven and Clark (2015) also gesture towards teachers feeling constrained in their pedagogical and text selection decisions due to curriculum and assessment pressures. The notion of constraints on teacher agency in the classroom also emerges in Frawley’s (2014) interviews with VCE English teachers, which revealed that a process model of writing was too challenging in the high-pressure, time-poor, VCE space. In a similar vein, Kitt’s (2019) interviews with teacher participants showed that VCE English teachers felt their approach to creative writing was limited by formulaic genres and rigid assessment pressures, and Roberts’s (2019) study of English leaders in government schools elucidated many of the complex and intersecting pressures of VCE English teachers/leaders, including data surveillance and heavy workload, illustrating a perpetuation of sentiments documented decades earlier in the aforementioned VCE Review of 1997 (State of Victoria, 1997). Furthermore, reflective practice has sought to foreground teachers’ voices in scholarship, as evidenced by Jordan’s (1999) musings on the efficacy of email for text discussion and McLean Davies’ (2009) consideration of critical theory for alternative text engagements.

Each of these studies, although enriching the discipline’s understanding of the demands and challenges of VCE English teachers, offer limited insights into the student experience. Etheredge’s (2003) study of memory work in relation to personal narrative writing in VCE English had the potential to speak to this gap in qualitative work on the student experience; however, by focussing on how teachers might scaffold students to demonstrate aptitude, it is difficult to discern the extent to which they sought to understand students’ perspectives and experiences of the task. Given the deeply heterogeneous nature of the subject and the diverse ways in which students and teachers mediate texts and curriculum in VCE English classrooms, knowledge of the subject will be furthered when teachers’ voices are placed in context and in conversation with the other voices which co-compose the VCE English assemblage: the students, the parents, faculty and school leaders, curriculum designers and more.

## Conclusions

This paper has presented an overview of the thematic and methodological trends which have dominated the scholarly and public debates on VCE English from 1990 to 2021. It found that considerable scholarly work has been undertaken in curriculum histories and commentaries concerned with external interference. Likewise, scholars have tended to focus on document or text-based work to extrapolate on teaching and learning, rather than attending to teaching and learning directly in situ. Finally, the review found that key stakeholders beyond teachers have yet to receive close attention in the research. Thus, one is left to wonder, where are the students? Why is it that researchers are yet to give sustained attention to the students of VCE English—their affective and intellectual experiences of the curriculum and texts, what they see as authentic and equitable learning activities and assessments?

There seems to be a strong correlation between the high-stakes, high-pressure context of senior secondary English and a lack of in situ research with teachers *and* students. Perhaps this is an effort of schools to protect their senior secondary students (and their academic results) from disruptions. Or perhaps this substantive gap in research is a result of the ideological and conceptual preoccupations noted above, fixating on texts and the intended curriculum. Moreover, when the general perception of rigidity—of transmission teaching—in this space is considered in tandem with the neoliberal aptitude ranking underscoring all senior secondary activities, one might understand how the student experience of VCE English can be seen as superfluous. But these conceptions need not define the subject and its future. With the release of the latest iteration of the VCE English Study Design in 2022, familiar media tropes and scholarly concerns with curriculum, assessment, texts and teachers will no doubt return, but researchers are also presented with a new opportunity to disentangle assumptions and disrupt repetitive patterns of inquiry—to take up fresh theoretical and methodological orientations that embrace the rich, unseen complexities and potentialities of the subject.

## Footnotes

1 Senior English in Victoria has always been contentious. For genealogies and discussions of senior English in Victoria prior to the accreditation of the VCE English course in 1990, see Green and Beavis (eds.) (1996).

2 For a more comprehensive account of the development of the VCE and politicisation (not specific to VCE English), including the complex interplay between curriculum and election cycles, opposition from the University of Melbourne and coverage in The Herald Sun and The Age, see Keating and Pannel, “The VCE media debate: 1989–1992”.

3 Although undertaken in an elective English subject (VCE Literature) with a different curriculum and narrower student cohort, Purcell’s (2021) exploration of affective moments in classroom praxis offers a model for scholars who might turn to affect to unsettle constructions of literary pedagogy in VCE English.
